# Trace element concentrations in livers of Common Buzzards *Buteo buteo* from eastern Poland

**DOI:** 10.1007/s10661-017-6135-8

**Published:** 2017-07-29

**Authors:** Ignacy Kitowski, Dariusz Jakubas, Dariusz Wiącek, Agnieszka Sujak, Grzegorz Pitucha

**Affiliations:** 1 State School of Higher Education in Chełm, Pocztowa 54, 22-100 Chełm, Poland; 2 Department of Vertebrate Ecology and Zoology, Faculty of Biology, University of Gdansk, Wita Stwosza 59, 80-308 Gdańsk, Poland; 3 Institute of Agrophysics, Polish Academy of Sciences, Doświadczalna 4, 20-290 Lublin, Poland; 4 Department of Biophysics, University of Life Sciences in Lublin, Akademicka 13, 20-933 Lublin, Poland; 5 Department of Zoology, Faculty of Biology and Agriculture, University of Rzeszow, Rejtana 16c, 35-959 Rzeszów, Poland

**Keywords:** Common Buzzard, Trace elements, Heavy metals, Liver, Poland

## Abstract

In this study, our aim was to determine the common sources of origin of 18 elements in the livers of Common Buzzards collected during the breeding season in an extensive agricultural landscape in south-east Poland with respect to age (adults and immatures) and sex (males and females). In all 34 specimens collected, the element concentrations followed the pattern of S > Na > Fe > Mg > Zn > Si > Cu > Mn > Ba > Se > B > Pb > Hg > Cd > Cr > Ni > Sr > V. Among the heavy metals examined, only the concentration of Pb was relatively high. Given the prevalence of farmland in the studied area (and the wide use of fertilizers), common use of lead-hunting ammunition and moderate concentration of Pb in fertilizers, the indirect influence of hunting ammunition ingested with food or as gastroliths was apparently responsible for the elevated levels of Pb in the livers of Common Buzzards. In our study, no significant sex-related differences were detected in the hepatic concentrations of any element. However, a significant age effect was observed for three elements, which had elevated levels in adults (Hg) and immature birds (B, Pb), and a significant age *x* sex interaction was found for S and Fe. These results might be explained by the importance of these elements in bone growth in immature birds (B), variable strategies of foraging between adults and immature birds (Pb), and possible intersex differences in the immature cohort in response to the presence of lead (S, Fe).

## Introduction

The relative proportions and interactions of trace elements in the organs of vertebrates, which include metals that are essential for life, influence the condition and functioning of individuals (McDowell 1992; Yang et al. 2008; Patlar et al. 2014). In birds, the concentrations of these essential elements depend on numerous biochemical, physiological, and ecological factors (Horai et al. 2007; Skoric et al. 2012; Ansara-Ross et al. 2013) and among the ecological factors, foraging areas, food composition, position in the trophic chain, and life-history stage and age are particularly important (Horai et al. 2007; Schummer et al. 2011; Skoric et al. 2012). When the concentrations of essential elements in an organism exceed threshold values, an individual may become intoxicated (Droual et al. 1991; Sileo et al. 2003; Kalisińska et al. 2008).

Raptors are the final consumers in food chains and therefore accumulate both trace elements, which are essential to their functioning, and contaminants from their prey, and because of their spatio-temporal relationships with their habitats, raptors can serve as models in studies of the ecological aspects of element accumulation in key organs (Ansara-Ross et al. 2013; Kim and Oh 2016). The Common Buzzard, *Buteo buteo*, occurs throughout Europe in a wide spectrum of breeding habitats, including agricultural zones, forests, suburban and urban areas, mountain regions, and wetlands (Cramp and Simmons 1980; Jedrzejewski et al. 1994). Common Buzzards, similarly to other raptors, are territorial and are opportunistic hunters of a wide variety of prey, including rodents, pigeons, weasels, and amphibians (Jedrzejewski et al. 1994; Goszczyński 1997; Goszczyński et al. 2005; Wuczyński 2003, 2005). Thus, the elements that accumulate in the organs of Common Buzzards may reflect contamination in mosaics of agricultural and forest habitats. The level of contamination in this species has been relatively well studied in western Europe (Hontelez et al. 1992; Battaglia et al. 2005; Jager et al. 1996; Licata et al. 2010; Castro et al. 2011; Carneiro et al. 2014), but studies from central and eastern Europe remain scarce and have often been based on small sample sizes and/or only focused on a single element (Houserova et al. 2007; Kalisinska et al. 2009).

The aims of the study were (1) to determine the concentrations of some essential elements (iron, manganese, zinc, selenium) and heavy metals (lead, mercury, cadmium) and then compare the concentrations with those of other species and reference values for toxicity and (2) to compare the hepatic concentrations of 18 elements (B, Ba, Cd, Cr, Cu, Fe, Hg, Mg, Mn, Na, Ni, Pb, S, Se, Si, Sr, V, and Zn) among individuals differing in age (adults vs. immature birds) and sex (males vs. females).

Considering the differences in the parental roles of females (incubation, chick rearing) and males (food provisioning) and the reverse sexual dimorphism in Common Buzzards (Cramp and Simmons 1980; Manosa and Cordero 1992), we expected to find differences between adult males and females in their exposure to contamination. Moreover, females may reallocate some toxic elements during egg formation, e.g., heavy metals from body tissues to eggshells (Burger 1994). Thus, considering that most of the birds in this study were collected during the breeding period, the concentrations of some heavy metals might have been lower in adult females. Furthermore, on the basis of age-related differences in hunting efficiency in raptors (Toland 1986; Ellis et al. 1993; Schindler 2002; Rutz et al. 2006), less experienced immature birds may hunt more frequently in suboptimal areas and focus on suboptimal prey, as compared with adults, and foraging on suboptimal prey (e.g., carrion) may make immature birds more susceptible to Pb contamination. Additionally, because Common Buzzards may feed on prey and carrion that is contaminated with Pb from hunting ammunition (Castro et al. 2011; Carneiro et al. 2014), we expected to find elevated Pb concentrations in at least some individuals, with the accompanying accumulation of other elements.

## Methods

### Origin of the studied birds

**Fig. 1:**
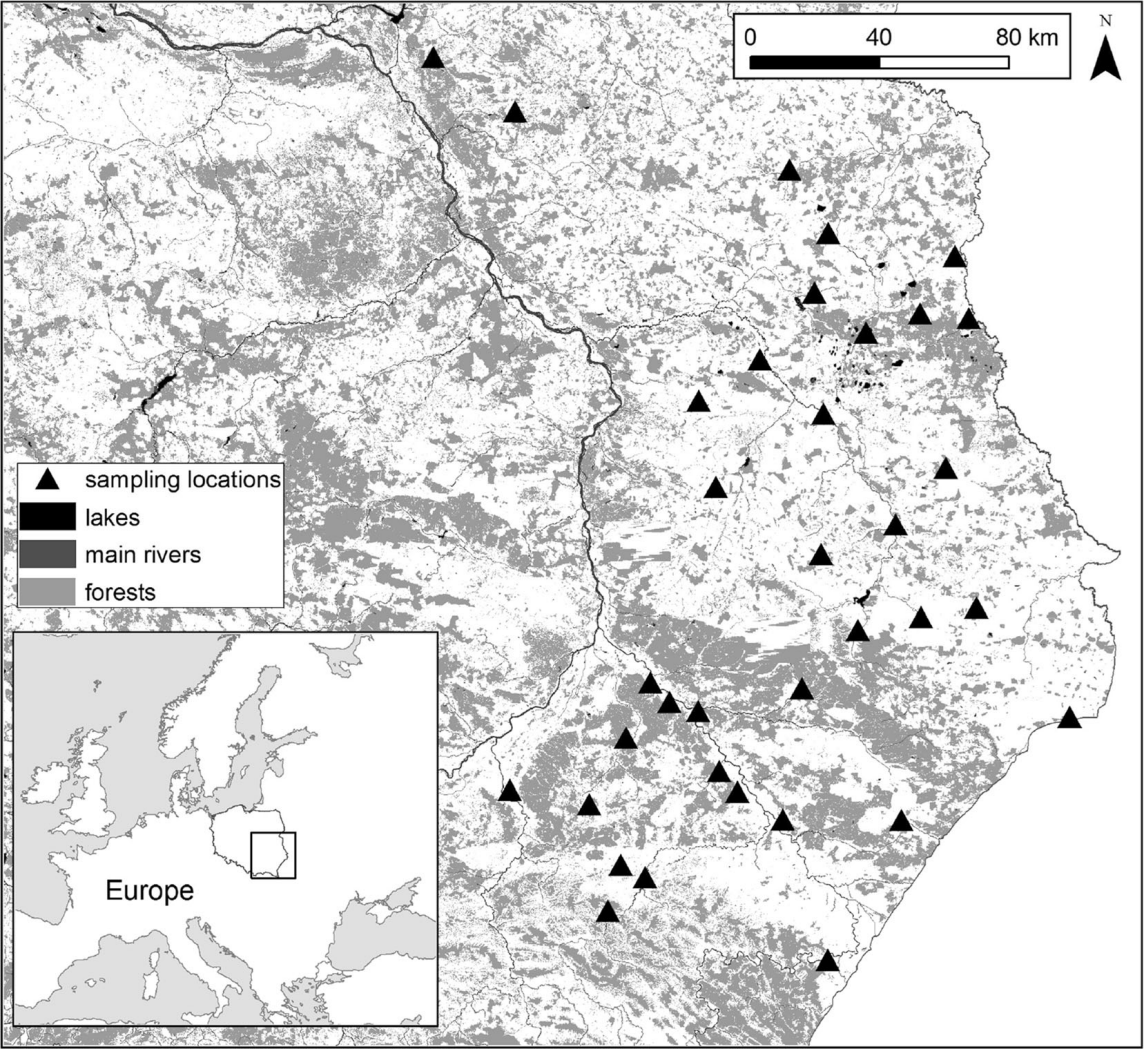
Study area with location of all sampling sites

In total, the livers of 34 Common Buzzards that were collected in an extensive agricultural landscape in south-east and central-east Poland (Lublin, Rzeszow, and Warsaw regions; Fig. 1) were analyzed. A large area of farmland with a low level of industrialization characterizes the study area. The livers were collected from dead individuals delivered to veterinary clinics in April–June between 2010 and 2014. The delivered birds were either dead or if they were found to be untreatable upon 134 delivery, they were euthanized by lethal injection by veterinary doctors to avoid unnecessary suffering. The birds did not remain in the clinics for more than 5 days. Some specimens (*N* = 5) were found during field observations under high-voltage power lines; their condition precluded any veterinary treatment. Another small group of specimens (*N* = 4) comprised road causalities. After being extracted from the bodies of the birds, the liver tissues were stored in freezers until analyses. Birds were sexed by internal examination after dissection and classified as either immature birds (≤2 years old) or adults (>2 years old) on the basis of their plumage, gonadal development, and iris color (Cramp and Simmons 1980; Baker 1993; Forsman 1999).

### Laboratory analyses

Before analyses, the livers were lyophilized and ground in a ceramic mortar. Before use, all glassware and instruments were rinsed with tap water, soaked in an acid bath (5 M HNO_3_) for 24 h, rinsed with demineralized water, and dried under a laminar flow hood to minimize the risk of metal contamination. Weighed portions of the samples (500 ± 1 mg) were mixed with 10 mL of concentrated HNO_3_ (Sigma Aldrich, Germany) and subjected to wet-ashing. Mineralization was conducted using a microwave digestion system, and optical temperature and pressure monitoring were performed on each sample during acid digestion (Berghof Speedwave) in Teflon vials (DAP 100 type) from Berghof GmbH, Eningen, Germany. The mineralization process was as follows: 15 min gradient from room temperature to 140 °C, 5 min at 140 °C, 5 min gradient from 140 to 170 °C, 15 min at 170 °C, and cooling to room temperature (varying times). The pressure did not exceed 12 bars throughout the entire mineralization process. After mineralization, a clear elemental solution was obtained that was cooled to room temperature and transferred to 50-mL volumetric flasks, which were filled with demineralized water (ELGA Pure Lab Classic). In this study, a Thermo Scientific iCAP Series 6500 ICP-OES (inductively coupled plasma optical emission spectrometer) equipped with a charge injection device (CID) detector was used for the elemental determinations. The spectrometer was controlled with the PC-based iTEVA software using the following instrumental parameters: RF generator power of 1150 W, RF generator frequency of 27.12 MHz, coolant gas flow rate of 16 L min^−1^, carrier gas flow rate of 0.65 L min^−1^, auxiliary gas flow rate of 0.4 L min^−1^, max integration time of 15 s, pump rate of 50 rpm, an axial viewing configuration, three replicates, and a flush time of 20 s.

The following multi-element stock solutions (Inorganic Ventures) were used as standards:

1. Analityk-46: ^63^Cu, ^57^Fe, and ^24^Mg in 5% HNO_3_-1000 mg L^-1^;
2. Analityk-47: ^75^As, ^111^Cd, ^52^Cr, ^208^Pb, ^55^Mn, ^201^Hg, ^60^Ni, ^45^Sc, ^79^Se, ^88^Sr, ^51^V, and ^66^Zn in 10% HNO_3_-100 mg L^-1^;
3. Analityk-83: ^40^Ca, ^39^K, ^24^Mg, ^23^Na, ^31^P, and ^32^S in 2% HNO_3_-1000 mg L^−1^

On the basis of the mineralization method (500 mg of sample diluted in 10 mL of HNO_3_ with a density of 1.51 g cm^−3^), the estimated Hg detection limit was 0.058 μg L^−1^ (3.72 × 10^−5^ μg/kg).

Samples (livers), including a blank (control) sample, were analyzed against a certified reference material, TraceCERT periodic table mix 1 for ICP (Fluka Analytical, Sigma Aldrich, Germany), to control the accuracy of the method under the laboratory conditions. To calculate the recovery percentage, three randomly selected samples were supplied with known amounts of the analytical standard and the mean percentage recoveries of the analyzed elements were calculated on the basis of the following equation: Recovery [%] = (C_E_/C_S_ × 100), where C_E_ is the experimental concentration determined from the calibration curve and C_S_ is the spiked concentration. All concentrations obtained in this study are presented as microgram per kilogram of dry weight (dw).

Background and toxicity levels (subclinical toxicity and moderate and severe clinical poisoning) of Cd and Pb were classified on the basis of data from the literature (Scheuhammer 1987; Guitart et al. 1994; Martin et al. 2008; Kim and Oh 2016).

### Statistical analyses

To compare the concentrations of particular elements among sexes and ages, two methods were used: for all elements simultaneously-PERMANOVA (non-parametric MANOVA based on the Bray-Curtis measure; Anderson 2001) with fixed factors (age and sex) and their interaction as explanatory variables; for particular elements-factorial analyses of variance (ANOVA) followed by a *post hoc* HSD test for unequal *N* (Sokal and Rohlf 1981).

The data set was tested for linearity and heterogeneity of variance using Q-Q plots (the quantile expected from a normal distribution vs. the quantile from the plot of observed residuals) and Levene’s test. Because data were not normally distributed, they were normalized using the following transformations: ln (Cd, Cu, Se), 1/x (Cr, Fe, Pb, V), and Box-Cox (B, Ni, Sr). The Hg data were ln (x + 1) transformed, because they contained zero values. Multivariate analyses were performed on log (x + 1) transformed data for all elements. The mean and SD are the values for particular groups presented in the tables. All statistical analyses were performed in STATISTICA 12.0 (StatSoft, Inc. 2014, USA) and PAST 3.0 (Hammer et al. 2001).

## Results and discussion

To our knowledge, this study is the first comprehensive investigation of inter-group differences in concentrations of hepatic elements in one of the most common bird of prey, the Common Buzzard, in central and eastern Europe. The following pattern of trace element concentration was found in the Common Buzzards: S > Na > Fe > Mg > Zn > Si > Cu > Mn > Ba > Se > B > Pb > Hg > Cd > Cr > Ni > Sr > V.

### Concentrations of selected heavy metals (lead, mercury, and cadmium)

The individuals accumulated from 0.43 to 17.2 μg/kg dw of Pb in the liver, but values that exceeded the background level of Pb (6–20 μg/kg dw) were classified as potentially toxic concentrations and were found in only 12% of individuals (Table 1). Similarly, other studies on Common Buzzards have found no individuals (South Korea; Kim and Oh 2016) or only 5% (Britain; Pain et al. 1995) of individuals with potentially toxic hepatic Pb concentrations. By contrast, 80% of juvenile Cinereous Vultures *Aegypius monachus* and 79% of adult Common Kestrels *Falco tinnunculus* from South Korea have been found to have potentially toxic liver Pb levels (Kim and Oh 2016). Strong industrialization in hunting territories might explain those results for young kestrels; whereas the relatively high levels of Pb in the livers of young vultures might have resulted from consumption of carrion containing lead shot. Hepatic concentrations of Pb in our study for adults and immature birds (mean: 2.84 and 1.19 μg/kg dw, respectively; Table 2) were higher than those of Common Buzzards in the Netherlands (0.7 μg/kg dw; Hontelez et al. 1992) and lower than those of buzzards in South Korea (5.56 μg/kg dw; Kim and Oh 2016). The low values recorded in the Netherlands may have been a consequence of a ban on the use of Pb-hunting ammunition enacted in the 1990s (Mateo 2009). Prey switching to species polluted by Pb (game species) might explain the relatively high mean Pb concentrations in our study. Over the past 20 years in Poland, the population dynamics of preferred Common Buzzard prey species common voles *Microtus arvalis* and tundra voles *Moeconomus oeconomus* have changed from regular cyclic fluctuations to chaotic irregularities, thus decreasing vole availability (Gliwicz 2010; Gliwicz and Jancewicz 2016). This decline in densities of preferred prey may have promoted intra-specific competition in Common Buzzards that resulted in a dietary shift from small mammals to alternative prey, such as game animals, medium-sized wild birds, moles, and fowl (Goszczynski et al. 2005), and with this shift, the probability of ingestion of Pb pellets from the tissues of shot game species may have increased. Avian species with more developed gizzards (e.g., pheasants, partridges, pigeons, and corvids) regularly feed on plant matter or animals with hard shells and regularly ingest sand or gravel grains (grit or gastroliths) to process food (Gionfrido and Best 1999). The ingestion of lead ammunition shot, which is mistakenly swallowed as natural grit, is a common pathway for Pb contamination in seedeaters, particularly galliformes (Fisher et al. 2006). Consequently, preying on medium-sized granivorous and herbivorous birds (which compose 40% of the diet of Common Buzzards in Poland; Goszczynski et al. 2005) with lead shot pellets in their muscles and/or gizzards may serve as primary source of Pb ingestion in the studied species.

**Table 1:** Background and toxic concentrations [μg/kg dw] of lead, cadmium and mercury in avian livers and their frequencies in livers of Common Buzzards (in brackets).

		Element concentration level [μg/kg dw]			
Element		Background	Subclinical toxicity	Moderate clinical poisoning	Severe clinical poisoning
Pb	Reference values	0–5 (A)	6–20 (B)	20–30 (B)	> 30 (B)
	Frequency in buzzards’ livers	30 (88.2%)	4 (11.8%)	–	–
Cd	Reference values	0–3.0 (C)	> 3.0 (C)	–	–
	Frequency in buzzards’ livers	33 (96.1%)	1 (2.9%)	–	–
Hg	Reference values	0–6.7 (D)	> 6.7 (D)	–	> 67.0 (D)
	Frequency in buzzards’ livers	33 (96.1%)	1 (2.9%)	–	–

Reference values are according to Martin et al. 2008 (A), Guitart et al. 1994 (B), Scheuhammer 1987 (C), and (D) Shore et al. (2011)

**Table 2:** Concentrations [μg/kg dw] of elements in adult and immature Common Buzzards. Only elements exhibiting a significant age effect (ANOVA, *p* < 0.05) are presented

	Adult (*N* = 20)		Immature (*N* = 14)		ANOVA
Element	Mean	SD	Mean	SD	*p* value
B	4.25	4.78	7.56	18.4	0.03
Hg	2.03	2.14	0.67	0.68	0.01
Pb	2.84	4.31	1.19	1.45	0.046

Hg is more toxic than other heavy metals because it has an extremely high affinity for the cysteines in proteins, and causes their irreversible inhibition (Quig 1998) and may lead to behavioral and reproductive disorders in many wild vertebrates (Clarkson and Magos 2006; Mutter et al. 2007). Hg compounds are primarily metabolized in the liver with the demethylation and conjugation with Se (Yang et al. 2008) or glutathione (Clarkson and Magos 2006). The liver, in contrast to the brain, is not a major target organ of Hg toxicity (Scheuhammer 1987; Scheuhammer et al. 1998).

Among the examined livers, the Hg concentration (8.47 μg/kg dw) in only one individual indicated subclinical toxicity (Table 1) (Shore et al. 2011). For the other samples, Hg concentration did not exceed the background level (Table 1).

The hepatic concentrations of Hg in our study for adult females and males (means: 1.83 and 2.31 μg/kg dw, respectively; Table 3) were similar to those reported for adult buzzards in Portugal (1.48 μg/kg dw) (Carneiro et al. 2014) and in the Czech Republic (2.6 μg/kg dw) (Houserova et al. 2007).

**Table 3:** Concentrations [μg/kg dw] of the elements in Common Buzzards

	AdFem (8)		AdMal (12)		ImFem (7)		ImMal (7)	
Element	Mean	SD	Mean	SD	Mean	SD	Mean	SD
B	3.10	1.65	5.03	6.01	12.7	26.0	2.43	1.28
Ba	5.32	0.32	5.39	0.45	5.45	0.36	5.02	0.35
Cd	0.63	0.41	1.29	0.94	0.88	0.80	0.78	0.46
Cr	0.43	0.08	0.48	0.10	0.46	0.06	0.64	0.40
Cu	17.4	7.83	18.6	9.27	20.1	8.28	14.3	3.10
Fe	1292	456.8	3031	4944	3190	3331	1102	271.9
Hg	2.31	2.04	1.83	2.26	1.09	0.72	0.25	0.25
Mg	724.8	123.9	812.4	90.3	786.6	97.4	746.8	98.6
Mn	11.3	4.19	11.0	2.69	11.5	3.43	7.93	2.74
Na	5080	699.7	4989	809.7	5203	607.6	4491	849.2
Ni	0.25	0.10	0.27	0.09	1.09	2.22	0.30	0.03
Pb	0.98	0.36	4.08	5.27	1.65*	1.99	0.73*	0.36
S	7548	777.3	8273	899.2	8581*	1052	7197*	842.9
Se	3.40	1.08	4.21	2.6	3.63	0.85	3.16	0.93
Si	40.2	10.1	41.1	13.6	34.7	6.06	35.1	8.55
Sr	0.43	0.33	0.50	0.48	0.67	0.49	0.65	0.20
V	0.13	0.03	0.19	0.16	0.18	0.17	0.16	0.07
Zn	99.2	19.1	105.5	15.7	105.6	18.1	93.6	18.3

Sample size is in parentheses. HSD test for unequal N is *p* < 0.05

*AdFem* adult females, *AdMal* adult males, *ImFem* immature females, *ImMal* immature males

*Significant differences between sex and age groups

Of the examined livers, one had a Cd concentration (3.90 μg/kg dw) indicating possible subclinical toxicity (Table 1). In the other liver samples, Cd concentrations did not exceed the background level (3.0 μg/kg dw), and mean concentrations of this element for the examined cohorts were within the range between 0.63 and 1.23 μg/kg dw (Table 1). This result was consistent with other previously published data in which only a small fraction of liver Cd concentrations ≥3.0 μg/kg dw have been found (Battaglia et al., 2005, Licata et al. 2010, Komosa et al. 2012, Carneiro et al. 2014).

Although Cu is an essential element in several biological processes, prolonged exposure to elevated concentrations may adversely affect organisms (Fuentealba et al., 2003; Mladenovic et al. 2014) by depleting fat and protein resources (Esselink et al. 1995; Ansara-Ross et al. 2013). The birds in this study accumulated 14.3–20.1 μg/kg dw of Cu (Table 3), a level consistent with those reported in other studies on Common Buzzards from the Netherlands and South Korea: 12.5–15.9 μg/kg dw (Jager et al. 1996; Kim and Oh 2016). Values higher than these have been detected only in studies from southern Italy (39.5 and 37.80 μg/kg dw Naccari et al. 2009; Licata et al. 2010) and may have resulted from the common use of antifungal herbicides containing Cu in olive orchards and vineyards and the subsequent accumulation of this element in local agrocenoses, particularly in the soil (Viti et al. 2008; Komarek et al. 2010). Most probably, a similar mechanism explains the elevated concentrations of Cu in Common Kestrels from agricultural areas in South Korea (Kim and Oh 2016).

### Concentrations of essential elements (iron, manganese, zinc, selenium)

The buzzards in this study accumulated an average of 1527 μg/kg dw of Fe in the liver. However, two individuals contained elevated (>3000 μg/kg dw) levels, an adult male (3881 μg/kg dw) and an immature female (4690 μg/kg dw), and two other individuals showed Fe hyper-accumulation, an immature female (10,215 μg/kg dw) and an adult male (18,502 μg/kg dw). In vertebrates, Fe is stored in various tissues and high concentrations of Fe in the liver are symptoms of advanced inflammatory states and exaggerated immunological responses (Grasman 2002; Weiss 2005), because heavy metals have pro-inflammatory properties (Kasperczyk et al. 2012). A high level of Fe in the liver may be associated with Cd, Pb, or Zn poisoning (Droual et al. 1991; Lewis et al. 2001). However, birds strongly infested with nematodes or fungi may accumulate large amounts of Fe (Weiss 2005; Kalisinska et al. 2008), thus potentially explaining the high level in some individuals with hyper-accumulated Fe. The levels of hepatic Fe in Common Buzzards vary among studies. As compared with our study (1527 μg/kg dw), both higher and lower concentrations have been reported in other studies with the same species: 2287 μg/kg dw in the Netherlands (Jager et al. 1996) and 987.0 μg/kg dw in South Korea (Kim and Oh 2016). Our results indicated that elevated levels of Fe might be more frequent (12% of 34 individuals) than previously assumed. To date, single raptors with elevated levels of Fe have been found; however, the birds have been in generally poor condition (Kalisinska et al. 2008, Kalisińska et al. 2006, Kim and Oh 2016). Such an increase in number of cases of buzzards with elevated hepatic levels of Fe might be connected to interactions primarily with Pb but also with Cd and Hg, although the correlation is weak (Spearman *r* = 0.40, *p* < 0.05) between hepatic concentrations of Pb and Fe.

Mn is essential trace element which supports the proper function and regulation of many biochemical reactions, particularly those related to development (McDowell 1992). The concentrations found in this study (7.93–11.39 μg/kg dw; Table 3) are similar to those reported for Common Buzzards in the literature (9.00–11.35 μg/kg dw; Jager et al. 1996; Naccari et al. 2009; Licata et al. 2010) and comparable to that reported for the White-tailed Eagle *Haliaeetus albicilla* from Poland (8.5 μg/kg dw; Falandysz et al. 2001).

The average hepatic Zn levels previously reported for Common Buzzards from Italy, Spain and South Korea, i.e., 121.8–144.0 μg/kg dw (Naccari et al. 2009; Licata et al. 2010; Perez-Lopez et al. 2008; Kim and Oh 2016), are higher than those recorded in the present study (93.6–105.6 μg/kg dw; Table 3). Additionally, the hepatic Zn concentration values in other raptors, namely, Japanese Sparrow Hawk *Accipiter gularis* (220 μg/kg dw), and Northern Goshawk *Accipiter gentilis* (184 μg/kg dw) (Horai et al. 2007), are considerably higher than those reported in this study. The transfer of large quantities of this metal to feathers and bones may explain these differences, because zinc is required for bone (Cook 2000) and feather formation, and large quantities of this metal are known to accumulate in bird feathers (Skoric et al. 2012; Ansara-Ross et al. 2013).

The hepatic Se levels found in this study were relatively low (3.16–4.21 μg/kg dw), a result consistent with the low levels of this element reported in the tissues of other vertebrates (Nowakowska et al. 2014) and with the reported deficit of selenium in soils in eastern Poland (Bombik et al. 2010; Chalabis-Mazurek and Walkuska 2014). Aquatic organisms accumulate relatively high levels of this element (Bergeron et al. 2010), but prey associated with aquatic habitats constitute only up to 5% of the biomass of the diet of the Polish Common Buzzard (Jedrzejewski et al. 1994; Goszczynski et al. 2005), thus indicating little opportunity for buzzards to obtain Se from this source.

### Inter-group differences

Multivariate analysis revealed that the concentrations of all elements together (PERMANOVA, Bray-Curtis similarity measure) were not significantly affected by age (*F*_1,30_ = −0.08, *p* = 1.00), sex (*F*_1,30_ = 0.21, *p* = 0.82), or the age *x* sex interaction (*F*_1,30_ = −1.39, *p* = 0.43). However, univariate analyses (ANOVA) performed separately for particular elements revealed significant inter-group differences for only five elements (Pb, S, B, Fe, and Hg). For the other elements, the effects were not significant (ANOVA, all *p* > 0.05). The contradictory results of multivariate and univariate analyses might be explained by the high overall similarity of most element concentrations, except for the five with differences. The lack of significant inter-group differences in the concentrations of many of the elements found in this study might be explained by similar dietary habits of the age and sex groups and the low rate of element excretion into eggs laid by females (Carneiro et al. 2014). Therefore, only the five elements significantly affected by sex, age, or the interaction are discussed.

The hepatic concentration of Pb was significantly affected by age (ANOVA, *F*_1,30_ = 4.33, *p* = 0.046) and the age *x* sex interaction (*F*_1,30_ = 5.81, *p* = 0.02). For the effect of age, adults had higher Pb concentrations than immature birds (Table 2) and for the sex interaction, immature females had higher values than immature males (HSD test for unequal *N*, all *p* = 0.03; Table 3). The concentration of Pb was not significantly affected by sex (*F*_1,30_ = 0.35, *p* = 0.56). The significant interaction effect might be explained by age-related differences in hunting efficiency and reverse sexual dimorphism (RSD). In many raptors, immatures are less efficient hunters than adults (Toland 1986; Ellis et al. 1993; Rutz et al. 2006), and RSD is more pronounced in adults, thus, resulting in females hunting for larger prey than males (Massemin et al. 2000; Kruger 2005). Both phenomena may lead to immature females feeding on suboptimal food, such as carcasses or easy-to-hunt wounded game mammals, and such a suboptimal diet may be exacerbated by a lack of optimal prey. Similarly to the results of our study, adult Common Kestrels from South Korea have been found to have higher hepatic Pb concentrations than those of immature birds (Kim and Oh 2016) and age differences have also been reported in owls, with higher hepatic Pb concentrations in adult Eagle Owls *Bubo bubo* (Kim and Oh 2016), and higher levels of Pb in the viscera of adult Little Owls *Athene noctua* (Zaccaroni et al. 2003), than in immature birds.

The hepatic concentration of S was significantly affected by the age *x* sex interaction (ANOVA, *F*_1,30_ = 11.23, *p* = 0.002), and immature females had higher values than immature males (HSD test for unequal *N*, all *p* = 0.03; Table 3). Neither age (*F*_1,30_ = 0.005, *p* = 0.95) nor sex (*F*_1,30_ = 1.10, *p* = 0.30) significantly affected the concentration of S. A difference in the intensity of the elimination of toxic elements among the individuals probably explained the differences in hepatic concentrations of S between immature females and males (Table 3). Hg, Cd, and Pb detoxification processes may be associated with high S demand because of sulfur amino acid involvement (Quig 1998, Miles et al. 2000, Tamas and Martinoia 2006, Netto et al. 2014, Toohey 2014). Hepatic concentrations of S are rarely studied in birds. The buzzards accumulated on average approximately twofold less sulfur (7197–8581 μg/kg dw; Table 3) than the White-tailed Eagles from northwest Poland (Falandysz et al. 2001). The values obtained within this study are close to the hepatic concentrations previously reported for White-tailed Eagles from eastern Poland, where as many as 36% of the individuals show subclinical toxicity and acute Pb clinical poisoning (Kitowski et al. 2017). This result may indicate that the buzzards, similar to the White-tailed Eagles (Kitowski et al. 2017), might have difficulty in obtaining the supply of S required for detoxification.

Boron influences the activity of many metabolic enzymes, the metabolism of steroid hormones, micronutrients (Ca, Mg), and vitamin D and may also play a role in improving arthritis, plasma lipid profiles, brain function, and antioxidant capacity (Devirian & Volpe 2003, Kurtoglu et al. 2005, Turkez et al. 2012). Boron supplementation increases bone regeneration and strength (Devirian and Volpe 2003, Uysal et al. 2009). These properties of B, particularly those referring to bones, are likely to be particularly important for the development of immature individuals, thus, explaining the significant effect of age on the hepatic concentration of B in the tested birds (ANOVA, *F*_1,30_ = 5.01, *p* = 0.03), with adults having lower concentrations than immature birds (Table 3). In our study, neither sex (F_1,30_ = 2.22, *p* = 0.15) nor the age *x* sex interaction (*F*_1,30_ = 1.03, *p* = 0.32) significantly affected B concentration.

The hepatic concentration of Fe was significantly affected by the age *x* sex interaction (ANOVA, *F*_1,30_ = 5.04, *p* = 0.03). The concentration of Fe was higher in immature females than males (Table 3), but *post hoc* analyses did not reveal any significant differences (HSD test for unequal *N*, all *p* > 0.19). Neither age (*F*_1,30_ = 0.16, *p* = 0.70) nor sex (*F*_1,30_ = 0.75, *p* = 0.39) significantly affected the Fe concentration. Intra-species differences in hepatic concentrations of Fe in birds, in contrast to interspecies differences (Beyer et al. 2004, Kim and Oh 2012, 2016), are very rarely discussed. Solely, the report of Kim and Oh (2016) has shown that, in contrast to our results, adult Common Kestrels from South Korea had higher hepatic Fe concentrations than immature birds. Differences between Fe levels in immature female and male buzzards might be connected to the response discussed above for Pb (Table 3).

Age significantly affected the hepatic concentration of Hg (ANOVA, *F*_1,30_ = 7.62, *p* = 0.01), and we observed its higher concentrations in adults than in immatures (Table 3). Neither sex (*F*_1,30_ = 3.22, *p* = 0.08) nor the age *x* sex interaction (*F*_1,30_ = 0.63, *p* = 0.43) significantly affected the Hg concentration. Other studies on Common Buzzards have confirmed the tendency for greater accumulation of Hg in the liver of adults, although these differences are not always significant (Houserova et al. 2005, Carneiro et al. 2014). Some results in contrast to our findings have also been reported (Castro et al. 2011).

The mean hepatic Hg concentration found in our study for adults was 2.03 μg/kg dw (Table 3), a value similar to the concentration found in an earlier study of adult Common Buzzards from Spain (the median for females and males was 1.96 μg/kg dw; Castro et al. 2011). In the present study, the concentration of Hg in livers of immature males was 0.25 μg/kg dw (Table 2), whereas considerably higher Hg concentrations have been reported for immature males of Common Buzzards from Spain (median 5.6 μg/kg; Castro et al. 2011) and for immature birds from Portugal (1.130 μg/kg dw; Carneiro et al. 2014). The relatively low levels of Hg found in our study might be partially explained by the diet of Common Buzzards in Poland, which primarily consists of rodents and medium-sized birds and a negligible contribution to the diet from passerines (Jedrzejewska and Jedrzejewski 1998; Goszczynski et al. 2005). Studies from east Poland on the Sparrow Hawk *Accipiter nisus* which has a larger contribution of passerines in its diet, have revealed generally high hepatic Hg levels (median 2.0 μg/kg dw; range 0.19–11.99 μg/kg dw) (Kitowski et al. 2016). Elevated levels of this heavy metal in granivorous passerines may be a result of the illegal use of Hg-dressed seeds in agriculture in Poland or neighboring countries (Kitowski et al. 2016).

### Possible sources of elements

The pattern of heavy metal concentration might be explained by habitat structure in the study area, which is characterized by a large area of farmland with a low level of industrialization. Thus, Cd and Pb may originate from agriculture, because those elements often contaminate fertilizers (Mortvedt 1995; Hooda 2010). Buzzards ingest both toxic elements with the tissues of their prey, i.e., small mammals and birds associated with agricultural landscapes. In Italy, fertilizers are recognized as the primary source of Cd contamination in Common Buzzards (Battaglia et al. 2005). Pellets from hunting ammunition may be the primary source of Pb contamination. Given moderate Cd concentrations and high Pb levels in the studied birds, the indirect influence of hunting ammunition ingested with food was apparently responsible for the elevated levels of Pb in Common Buzzards. Although Hg is recognized as a common contaminant in fertilizers (Mortvedt 1995; Zhao and Wang 2010), in eastern Poland, coal combustion, commonly used for heating (Stala-Szlugaj 2011; Central Statistical Office 2015), is a considerable source of Hg emissions (Hlawiczka and Cenowski 2013). Thus, two possible sources might have contributed to Hg contamination in the livers of Common Buzzards in this study: a direct source from atmospheric emissions and an indirect source from agriculture through consumption of prey. Regardless of the source, the levels of Hg contamination were comparable to those in birds from other parts of Europe.

## Conclusions

The absence of inter-group differences for age and sex in the concentrations of many of elements found during our study might be explained by the high similarity of diets and the low rate of element excretion into the eggs laid by females. Among the toxic elements examined, the hepatic concentration of Pb was relatively high. Given the prevalence of farmland in the study area (and wide use of fertilizers), common use of lead-hunting ammunition and moderate concentration of Pb from fertilizers, the indirect influence of hunting ammunition ingested with food or as gastroliths was apparently responsible for the elevated levels of Pb in the livers of Common Buzzards. Less experienced individuals feeding on suboptimal foods (carrion, wounded game species) might explain the elevated levels of Pb in immatures. Compared with those in other studies, low hepatic concentrations of Hg and Se might indicate low contributions of granivorous passerines and amphibians contaminated by these heavy metals in the diets of the studied birds.
